# Alcohol in Diet

**Published:** 1903-02-21

**Authors:** 


### Alcohol in Diet.

According to a letter from the Paris correspondent of the *Times*, which appeared in the issue of the paper for the 14th instant, a number of eminent physicians and chemists across the channel are engaged in a somewhat belated controversy respecting the alimentary value of alcohol, and are arriving at differing conclusions in which the differences seem to be chiefly verbal, and to turn upon the question whether the term alimentary substance should be restricted to one which is capable of contributing to the growth of tissue, or extended to one which by its combustion supplies heat transformable into energy. As thus stated, the dispute might with propriety be referred to a maker of dictionaries, even if he were no better than the "harmless drudge" described by Dr. Johnson; for we apprehend that, as to the transformation of alcohol in the system, there is at present no room for doubt. In the words of Dr. Binz, "it is very probable that alcohol is completely oxidised into carbonic acid and water during the process of assimilation; at least no other secondary products resulting from its disintegration have as yet been detected." It is well known that the conclusions arrived at by Lallemand, Perrin, and Duroy have long since been shown to be entirely erroneous.

The truth is, moreover, that any possible discussion concerning the dietetic value of "alcohol" is at once rendered futile by its vagueness. It is like a discussion on the therapeutic value of "medicine," in which no distinction was drawn between digitalis and senna. Nobody drinks pure alcohol; and the numerous drinks which contain alcohol contain so many other and such widely different ingredients that no common measure for their operation is attainable. The *Times* tells us that M. Duclaux, of the Pasteur Institute, who has taken a leading part in the controversy, declares that a litre of "wine" per day may be safely or profitably consumed by a person of average constitution, while Professor Joffroy would limit the daily consumption to three-quarters of a litre, and declares that the "wine" should not contain more than 10 per cent. of alcohol. The condition would exclude port, madeira, sherry, champagne, and all the better varieties of burgundy, leaving little but poor burgundy, poor Rhine wine, claret, and moselle, all of them liquids of which, speaking as a matter of personal taste, we would rather be limited to three quarters of a litre than compelled to drink a whole one. The saccharine contents of grape juice are well known to range from about 13 to about 30 per cent., with corresponding differences in the amount not only of the alcohol which is immediately yielded by fermentation, but also in the amount and kind of the œnanthic and other ethers which are developed in course of time. Moreover, especially in wines "made in Germany," inferior grape juice is regularly "doctored" by the addition of extraneous sugar, which, unless some still cheaper method has recently been discovered, is obtained by the action of commercial sulphuric acid upon commercial starch. In pure wine, according to a well-known authority, we may expect to find, before adulteration, various alcohols, but chiefly the ethylic, several compound ethers, appreciable quantities of tartaric, racemic, malic, succinic, glycolic, oxalic, acetic, and tannic acids, together with glycerine, a trace of acetaldehyde, and traces of butyric, propionic, and œnanthic acids. Adulteration, in addition to innocuous vegetable colouring matters, such as the juice of mulberries or bilberries, supplies poisonous coal-tar colours, and calcium sulphate, together, in all probability, with a certain amount of arsenic derived from the sulphuric acid used in the manufacture of cheap sugar. It is tolerably evident that to speak of the general result as "wine," and to say how much of it may be consumed with advantage or with impunity, is mere playing with words.

In this country, at least, we think it will be the general opinion of the medical profession that even Professor Joffroy has been too liberal in his estimate of quantity. A litre, it must be remembered, equals 35 fluid ounces, so that three-quarters of a litre amounts to a good deal more than an imperial pint. It is part of the French discussion to admit that drunkenness is steadily on the increase among our neighbours, and that the diseases consequent upon it are increasing in a corresponding ratio. It may be, therefore, that the amount of the allowance suggested by the French savants is a concession to the hardness of heart of their people, like Moses's concession of facilities for divorce; for M. Duclaux's suggested allowance of "spirit," twelve to fifteen liqueur glasses a day, strikes us as being more excessive even than his allowance of wine. We have never numbered ourselves among the fanatical opponents of a moderate consumption of alcohol; but we can entertain no doubt that the generally improved health of our people is very largely due to the greater moderation in drinking which has become characteristic of all classes but the very lowest. The Registrar-General has just published the statement that the mortality of England and Wales for the year 1902 has been only 16·3 per thousand—a rate never before attained among ourselves, and which has never even been approached in any other country. It is one of which we may be justly proud, and which we should by all practicable means endeavour to maintain.

